# Identification of DNA methylation biomarkers for evaluating cardiovascular disease risk from epigenome profiles altered by low-dose ionizing radiation

**DOI:** 10.1186/s13148-024-01630-0

**Published:** 2024-02-01

**Authors:** Jihye Park, Hae-June Lee, Yu Kyeong Han, Keunsoo Kang, Joo Mi Yi

**Affiliations:** 1 Department of Microbiology, Dankook University, Cheonan, 31116 South Korea; 2 Division of Radiation Biomedical Research, Korea Institute of Radiological and Medical Sciences, Seoul, 01812 South Korea; 3 Department of Microbiology and Immunology, College of Medicine, Inje University, Busan, 47392 South Korea

**Keywords:** DNA methylation, Low-dose radiation, Human aortic endothelial cells (HAECs), Methylation biomarker, Assessment of cardiovascular risk

## Abstract

**Background:**

Environmental exposure, medical diagnostic and therapeutic applications, and industrial utilization of radionuclides have prompted a growing focus on the risks associated with low-dose radiation (< 100 mGy). Current evidence suggests that such radiation can induce epigenetic changes. Nevertheless, whether exposure to low-dose radiation can disrupt endothelial cell function at the molecular level is unclear. Because endothelial cells play crucial roles in cardiovascular health and disease, we aimed to investigate whether low-dose radiation could lead to differential DNA methylation patterns at the genomic level in endothelial cell (EC) lines.

**Methods:**

We screened for changes in DNA methylation patterns in primary human aortic (HAECs) and coronary artery endothelial cells following exposure to low-dose ionizing radiation. Using a subset of genes altered via DNA methylation by low-dose irradiation, we performed gene ontology (GO) analysis to predict the possible biological network mediating the effect of low-dose radiation. In addition, we performed comprehensive validation using methylation and gene expression analyses, and ChIP assay to identify useful biomarkers among candidate genes for use in detecting low-dose radiation exposure in human primary normal ECs.

**Results:**

Low-dose radiation is sufficient to induce global DNA methylation alterations in normal EC lines. GO analysis demonstrated that these hyper- or hypo-methylated genes were linked to diverse biological pathways. Our findings indicated a robust correlation between promoter hypermethylation and transcriptional downregulation of four genes (*PGRMC1*, *UNC119B*, *RERE*, and *FNDC3B*) in response to low-dose ionizing radiation in HAECs.

**Conclusions:**

Based on these findings, the identified genes can serve as potential DNA methylation biomarkers for the assessment of cardiovascular risk upon exposure to low-dose radiation.

**Supplementary Information:**

The online version contains supplementary material available at 10.1186/s13148-024-01630-0.

## Background

The health risks of low-level exposure to ionizing radiation (IR) are thought to be primarily related to several types of cancer in directly exposed populations including survivors of the atomic bomb explosion in Japan [1–3]. Radiation-induced heart disease as a consequence of direct damage caused by high-dose thoracic radiotherapy has been acknowledged for several decades [4, 5]. The increased emphasis on low-dose radiation risks has grown with environmental, medical diagnostic, therapeutic exposures, and industrial applications of radiation, prompting attention to these concerns [6, 7].

According to the guidelines of the International Commission on Radiological Protection (ICRP), individuals susceptible to recurrent radiation exposure risk, such as healthcare and nuclear industry workers, are commonly subject to monitoring and limitations on effective doses. The recommended threshold is 100 mSv every 5 years (equivalent to 20 mSv per year), with a maximum allowance of 50 mSv in any given year [8, 9]. The estimation of risks associated with detrimental effects of exposure to low-dose radiation (LDR) was done by extrapolating data derived from high-dose radiation (HDR) exposure, employing a linear model without a threshold. Accumulating evidence indicates that living organisms, including humans, may exhibit different responses to low-dose radiation (LDR) compared to high dose radiation(HDR) [10]. There is little doubt that intermediate and high doses of ionizing radiation (> 100 mSv), administered acutely or over an extended period, lead to adverse effects in humans, including the development of cancer. Brenner et al. provided a list of approximate mean doses relevant to societal low-dose radiation exposures and low-dose radiation risk estimation (ranging from 3 to 30 mSV) in most radiological examinations [6]. Understanding the implications of low-dose radiation remains socially relevant and encompasses various issues, including cancer screening tests, the future of nuclear power, occupational radiation exposure, manned space exploration, and concerns related to radiological issues.

The Life Span Study conducted on Japanese atomic bomb survivors presented evidence for an increased risk of cardiovascular disease at lower dose levels, specifically below 5 Gy, and with mean doses significantly less than 0.5 Gy [11, 12]. The Life Span Study data, however, did not reveal any notable nonlinear association of the radiation dose–response with cardiovascular disease mortality. However, the specific form of the dose–response relationship, especially at doses below 0.5 Gy, remains uncertain [12]. Therefore, the magnitude of cardiovascular disease risk remains uncertain at low doses of radiation (< 0.1 Gy), typically encountered in medical diagnostic exposures. Although experimental and epidemiological evidence has established a relationship between exposure to low-dose IR and the development of solid cancers and leukemia, the relationship between the long-term risk of cardiovascular disease and low-dose radiation exposure remains unclear [13].

Endothelial cells (ECs) play a pivotal role in the cardiovascular system. However, the presence of cardiovascular risk factors diminishes their function. Because of their strategic anatomical location between the circulating blood and the vessel wall, ECs actively regulate vascular structure and function. EC dysfunction is a crucial starting point for various types of circulatory diseases [14]. The heart is considered the most etiologically relevant target tissue for ischemic heart disease, and the dose to the heart is often used in the analysis of radiation-induced ischemic heart disease [5]. The critical role of vascular ECs in circulatory diseases suggests that large arteries (e.g., the aorta and carotid) may also be etiologically relevant targets.

Epigenetic mechanisms are adaptable genomic parameters capable of altering genome function in response to environmental effects [15]. Additionally, they offer a mechanism to reliably propagate gene activity states from one generation of cells to the next [16]. Epigenetic events are recognized for their role in regulating gene expression during development and differentiation and in response to environmental stimuli, including IR [15]. Epigenetic mechanisms regulate gene expression mainly through DNA methylation, histone modification, and alteration of nucleosome positions along the DNA [17]. An increasing body of evidence suggests that epigenetics offers significant potential for developing biological markers to predict the vulnerability of subjects exposed to specific risks and identify individuals more susceptible to developing diseases [18]. Recently, the biological or genome-wide impact of IR has sparked increasing interest in epigenetic alterations [19]. In previous studies, we showed that IR induces hypomethylation in several types of cancers [20, 21]. In addition, although there is a general agreement on the negative impact of high doses of radiation and on the mechanisms of cell injury, the biological effects and mechanisms of the response to low-dose IR, including through exposure to diagnostic imaging, remain poorly understood.

To understand the epigenetic impact of low-dose IR, in this study, we explored its influence on global genome-wide methylation profiles in normal EC lines; the Illumina HumanMethylationEPIC BeadChip array platform was employed for methylation profiling, encompassing 99% of the RefSeq genes [22]. This technology enables the comprehensive analysis of differential global DNA methylation. This study provides valuable insights into the DNA methylation changes induced by low-dose radiation in normal ECs, offering potential methylation biomarkers to assess the risk of radiation exposure and diseases, including cardiovascular disease.

## Materials and methods

### Cell culture, drug treatment, and irradiation

Human aortic endothelial cells (HAECs) and human coronary artery endothelial cells (HCAECs) were purchased from Lonza Group Ltd. (Walkersville, MD, USA) and cultured in endothelial growth medium-2 microvascular medium (Lonza) at 37 °C and 5% CO_2_ in a humidified incubator.

### Irradiation

We seeded 5 × 10^5^ HAECs or HCAECs in 100 mm culture dishes. The cells were exposed to gamma (*γ*) rays through the “aircenter” mode after refreshing the culture medium. For LDR radiation, cells were exposed to radiation in a CO_2_ incubator at a dose rate of 6 mGy/h for 0.1 (~ 16.7 h) or 0.5 Gy (~ 83.3 h) with a ^137^Cs (370 GBq) irradiator (Chiyoda Technol Corp., Tokyo, Japan) in a facility specialized for low-dose radiation at the DIRAMS, South Korea. For HDR, ranging from 2 to 8 Gy, the cells were also exposed to radiation at a dose rate of ~ 2 Gy/min using a BIOBEAM 8000 (Gamma Service Medical GmbH, Germany) with a 77.33TBq ^137^Cs source at room temperature. For experimental analysis, the cells were harvested 48 h after the completion of irradiation.

### HumanMethylationEPIC BeadChip array analysis

Genome-wide DNA methylation levels at approximately 850,000 CpG sites were determined using the Infinium HumanMethylationEPIC BeadChip Kit (Illumina), according to the manufacturer’s instructions. Raw data were analyzed using the Minfi package in R [23]. The degree of DNA methylation at each CpG site was represented by beta (*β*)-values, which are used to estimate the methylation level and range from 0 (unmethylated) to 1 (fully methylated). To identify the differentially methylated CpG sites between the control and test groups, we discarded sites with *β*-values < 0.1. CpG sites located within 1500 bp of the transcription start site (TSS) were used in this study.

### Gene ontology analysis

The potential functions of genes proximal to the differentially methylated CpG sites were inferred using Metascape (https://metascape.org/gp/index.html) [24]. The top 500 CpGs, either hypermethylated or hypomethylated, exhibited the largest differences in *β*-values between the control and test groups.

### Network analysis

Network analysis of selected target genes was performed using the STRING database (https://string-db.org/). STRING functions as both a database and a visualization platform, offering insights into protein–protein interactions.

### Methylation-specific PCR

DNA was extracted from HAECs and HCAECs following the standard phenol–chloroform extraction. Bisulfite modification of genomic DNA was performed using the EZ DNA Methylation Kit (Zymo Research). For methylation-specific PCR (MSP) and quantitative methylation analyses, we referred to a previously described procedure [25]. The primer sequences used are listed in Table S1 (Additional file 1).

### Bisulfite sequencing

One microgram of genomic DNA from each sample was bisulfite-converted using the EZ DNA Methylation Kit (Zymo Research), following the manufacturer’s protocol. Bisulfite-modified DNA was PCR-amplified, gel-purified, and subcloned into the pCRII-TOPO vector (Invitrogen). At least five to seven clones were randomly selected and sequenced on an ABI3730xl DNA analyzer to ascertain the methylation patterns of each locus. The primer sequences used for bisulfite sequencing are listed in Table S1 (Additional file 1).

### Quantitative real-time reverse transcription PCR

Total RNA was isolated from the control and irradiated samples of HAECs and HCAECs using TRI-Solution (BioScience Technology) following the manufacturer’s protocol. RNA quantity was measured using a NanoDrop 2000/2000c instrument (Thermo Fisher Scientific), and 1 μg of total RNA was reverse-transcribed into cDNA using the iScript™ cDNA Synthesis Kit (Bio-Rad). For the expression studies, primers were designed using the Primer3 web tool (http://frodo.wi.mit.edu/primer3) and are listed in Table S1 (Additional file 1). Quantitative real-time reverse transcription PCR (qRT-PCR) was performed on a CFX96™ Real-Time PCR Detection System (Bio-Rad) using SYBR Green Master Mix (Thermo Fisher Scientific). The expression levels of target genes were normalized against actin levels, and all relative quantifications of expression levels were calculated using the ∆∆C_t_ method.

### Western blot analysis

The cells were lysed in a lysis buffer. Equal amounts of total protein were loaded onto 4–12% SDS–PAGE gels and transferred onto PVDF membranes (GE Healthcare Life Sciences). The membranes were blocked with 5% skim milk dissolved in TBS containing 0.02% Tween 20 and incubated overnight at 4 °C with specific primary antibodies. The membranes were subsequently incubated with horseradish peroxidase-conjugated secondary antibodies. Protein bands were visualized using the Fusion FX5 system (Vilber Lourmat). The following primary antibodies were used: anti-DNMT1 (Abcam), anti-DNMT3A (Cell Signaling Technology), anti-DNMT3B (Santa Cruz), anti-DNMT3L (Santa Cruz), anti-MBD4 (Santa Cruz), anti-MBD2 (Santa Cruz), anti-MeCP2 (Santa Cruz), and anti-Actin (Proteintech).

### Chromatin immunoprecipitation analysis

Chromatin immunoprecipitation (ChIP) assay was performed as described previously [26]. PCR was performed using a C1000 Thermal Cycler (Bio-Rad), and the ChIP primers are listed in Table S1 (Additional file 1). An anti-DNMT1 antibody (Invitrogen) was used for the immunoprecipitation of DNMT1-associated chromatin fragments.

### Statistical analysis

Statistical analyses were performed using the GraphPad Prism software version 9.0 (https://www.graphpad.com). The results are presented as the mean ± standard deviation (SD). Student’s *t* test was performed to calculate statistical significance. *P* values < 0.05 were considered to indicate significant differences.

## Results

### Global DNA methylation changes induced by IR in HAECs and HCAECs

**Fig. 1:**
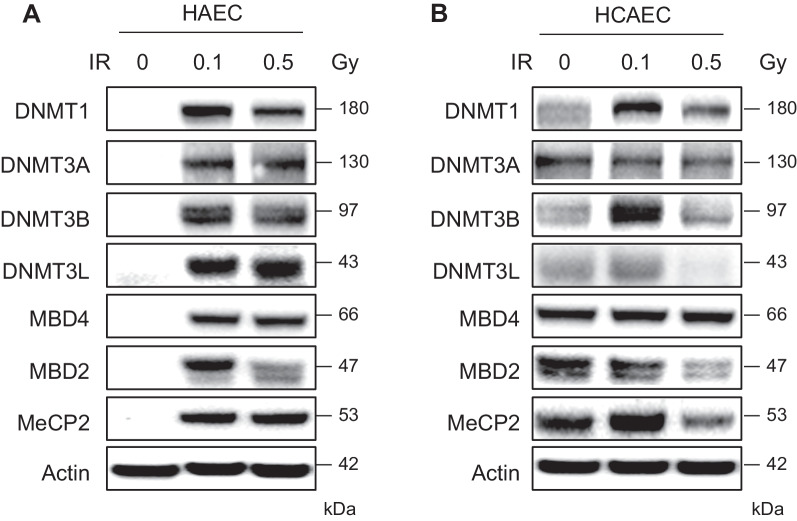
Low-dose radiation induces changes in the levels of epigenetic regulators in HAECs and HCAECs. **A**, **B** Western blots showing the protein expression levels of methylation regulatory factors (DNMT1, MBD2, MBD4, and MeCP2) in HAECs and HCAECs irradiated with 0.1 and 0.5 Gy and a control. The blots were probed using anti-DNMT1, anti-DNMT3b, anti-DNMT3A, anti-DNMT3L, anti-MBD4, anti-MBD-2, and anti-MeCP2 antibodies. Actin expression was used for normalization

To study the effects of low-dose radiation on DNA methylation patterns across the genome, we selected HAECs and HCAECs. Initially, we investigated the impact of IR treatment on normal HAECs and HCAECs to assess its influence on the expression of established regulators of DNA methylation, including DNA-methyltransferase 1, 3A, 3B, and 3L (DNMT1, DNMT3A, DNMT3B, and DNMT3L), methyl-CpG-binding domain proteins 2 and 4 (MBD2 and MBD4), and methyl CpG-binding protein 2 (MeCP2). Western blot analyses revealed a notable increase in the protein levels of numerous DNA methylation regulatory factors in cells exposed to varying IR doses (2, 4, 6, and 8 Gy), suggesting that IR treatment induces alterations in global DNA methylation (Additional file 1: Fig. S1). Nonetheless, we focused on investigating whether low-dose IR could induce global DNA methylation changes in HAECs and HCAECs. Therefore, we examined protein expression levels in cells exposed to low-dose IR (0.1 and 0.5 Gy) using western blot analysis. A significant increase was observed in the protein levels of most of the DNA methylation regulatory factors in cells subjected to 0.1 Gy irradiation. This finding suggests that low-dose IR is potent enough to induce global DNA methylation alterations in both HAECs and HCAECs (Fig. 1).

### Genome-wide DNA methylation profiles induced by low-dose IR in HAECs and HCAECs

To evaluate the actual DNA methylation changes at the genome-wide level in HAECs and HCAECs, we used the HumanMethylationEPIC BeadChip platform developed by Illumina. This advanced platform targets 850,000 CpG sites in the most biologically relevant regions of the human methylome. The methylation levels at individual CpG sites were quantified as continuous variables. Using this platform, we performed an extensive investigation of distinct DNA methylation patterns in response to varying low-dose radiation levels (0.1, 0.5, 2, and 6 Gy) in two separate EC types.

**Fig. 2:**
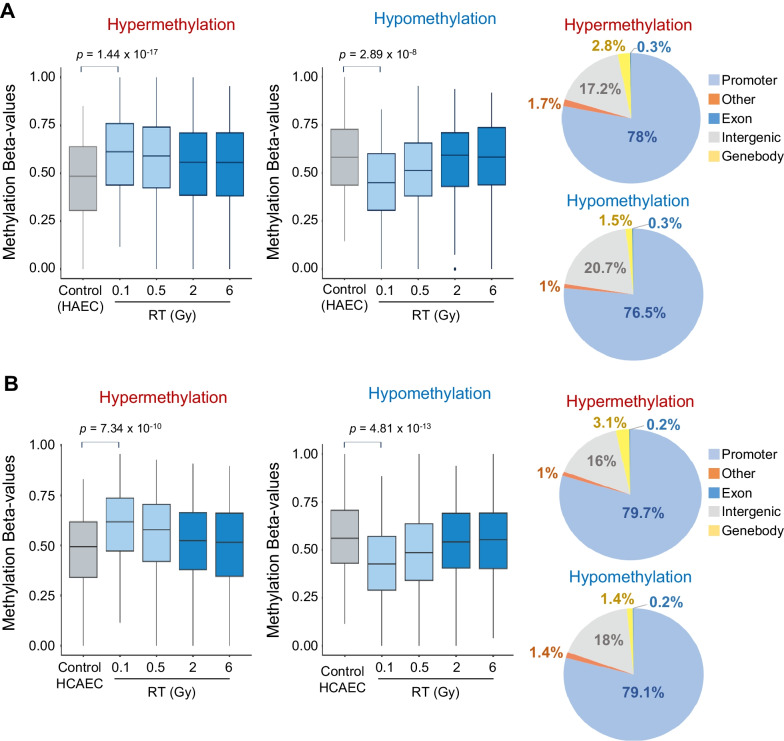
Genome-wide differentially methylated regions in irradiated HAECs and HCAECs. **A**, **B** Genome-wide DNA methylation levels were compared between irradiated HAECs and HCAECs and control using the HumanMethylationEPIC BeadChip platform. A total of 1064 (HAECs) and 483 (HCAECs) CpG sites were hypermethylated, whereas 392 (HAECs) and 573 (HCAECs) CpG sites were hypomethylated. *P* values were calculated using the Mann–Whitney *U* test. The pie chart illustrates the distribution of genome-wide coverage among differentially methylated regions. The promoter, intergenic region, gene body, exons, and other regions are shown

We identified substantial alterations in DNA methylation levels among irradiated HAECs and HCAECs compared to that in the controls. These changes encompassed both hypermethylation and hypomethylation events, with statistical significance confirmed using an adjusted *P*-value threshold of 0.001 (Benjamini–Hochberg correction). To explore the differential DNA methylation patterns between irradiated HAECs or HCAECs and controls, we implemented *β*-value = 0.1 as a filtering cut-off criterion. We detected 1064 hypermethylated and 392 hypomethylated probes, signifying distinct methylation patterns in irradiated HAECs, specifically at a low dose (0.1 Gy), compared to the controls (Fig. 2A). In addition, we detected 482 hypermethylated and 573 hypomethylated probes in the irradiated HCAECs (Fig. 2B). To gain insights into the overall distribution of these hypermethylated and hypomethylated loci, we analyzed their occurrence within the promoter, intergenic region, gene body, exons, and other regions (Fig. 2).

**Fig. 3:**
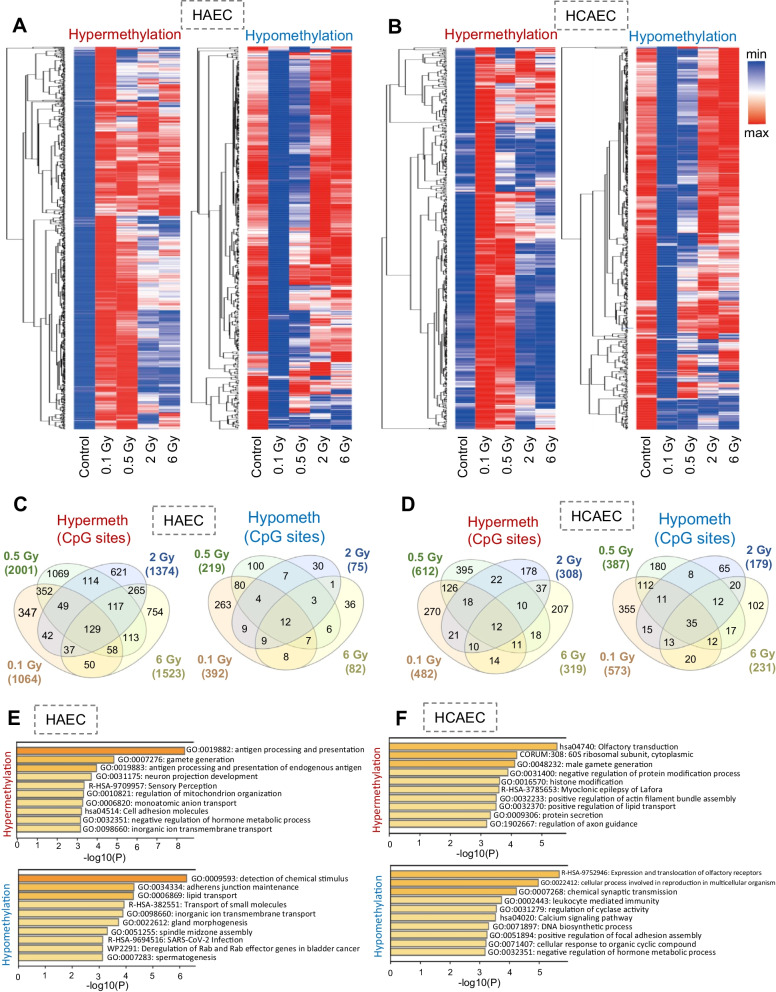
Differentially methylated genes in irradiated HAECs and HCAECs. Heat map and hierarchical clustering dendrogram of differential gene methylation profiles from irradiated **A** HAECs and **B** HCAECs. **C**, **D** A Venn diagram illustrates the number of hypomethylated and hypermethylated CpG sites identified among four distinct radiation doses, including a low dose. **E**, **F** GO analysis of genes associated with differentially methylated CpGs in HAECs exposed to low-dose radiation (0.1 Gy). The GO analysis was performed using Metascape, based on the genes derived from differentially methylated CpGs in **E** HAECs or **F** HCAECs irradiated with low-dose radiation (0.1 Gy) compared to controls

Promoter regions were delineated as spanning 1500 bp upstream of the transcriptional start site (TSS), encompassing exon 1. Intergenic regions were defined as genomic areas that did not fall within the scope of other specified classes. In the irradiated HAECs, differentially methylated regions were distributed across various gene regions in the following order: promoter region (78%) > intergenic region (17.2%) > gene body (2.8%) > other and exon (2%) for hypermethylated regions, and promoter region (76.5%) > intergenic region (20.7%) > gene body (1.5%) > other and exon (1.3%) for hypomethylated regions (Fig. 2A). In the methylation profiles of irradiated HCAECs, differentially methylated regions were located in multiple gene regions and ordered as follows: promoter region (79.7%) > intergenic region (16%) > gene body (3.1%) > other and exon (1.2%) for hypermethylated regions, and promoter region (79.1%) > intergenic region (18%) > gene body (1.4%) or other and exon (1.6%) for hypomethylated regions. Previous studies have highlighted the significant role of DNA methylation within the gene body in regulating transcription [27] (Fig. 2B). Subsequently, we employed more stringent criteria, requiring a hypermethylation fold-change > 3 and a hypomethylation fold-change < 3 in irradiated HAECs or HCAECs compared to the methylation profiles of controls. Hierarchical clustering revealed 1064 CpG sites with differential methylation patterns, comprising 1064 hypermethylated and 392 hypomethylated sites in HAECs and 482 hypermethylated and 573 hypomethylated sites in HCAECs (Fig. 3A, B). These findings suggest that exposure to low-dose radiation can lead to global DNA methylation profile changes in HAECs and HCAECs.

### Functional prediction of DNA methylation changes in HAECs or HCAECs treated with low-dose radiation

A comparison of the differential CpG sites in HAECs or HCAECs irradiated with various doses demonstrated that 129 hypermethylated genes for HAECs and 12 for HCAECs and 12 hypomethylated genes for HAECs and 35 for HCAECs were shared among the four radiation doses (0.1, 0.5, 2, and 6 Gy), with each dose of radiation possessing its own set of unique genes (Fig. 3C, D). To understand the functional significance of hypermethylate.

d CpG sites in low-dose-irradiated HAECs and HCAECs, we conducted a gene ontology (GO) analysis using Metascape (Fig. 3E, F). We conducted a GO analysis of the identified hypermethylated CpG sites in 0.1 Gy-irradiated HAECs and HCAECs using the Kyoto encyclopedia of genes and genomes (KEGG) database, which covers a wide range of biological processes, molecular functions, and cellular components. Hypermethylated or hypomethylated CpG sites in low-dose-irradiated HAECs and HCAECs were associated with several pathways, including antigen processing and presentation, detection of chemical stimuli, olfactory transduction, and expression and translocation of olfactory receptors (Fig. 3E, F). Therefore, our data imply that hyper- or hypomethylated genes or CpG sites influenced by low-dose radiation may contribute to potential biological networks, indicating that low-dose irradiation can affect normal ECs at the molecular epigenome level.

### Correlation between promoter methylation and transcriptional expression of candidate genes in irradiated HAECs

**Fig. 4:**
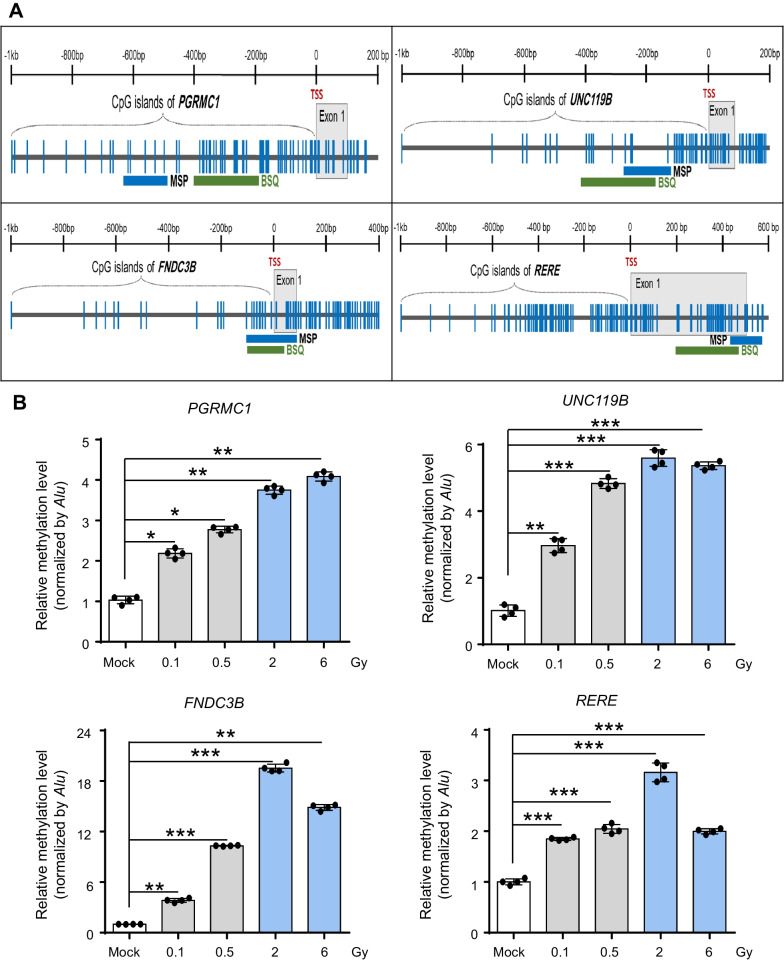
Promoter DNA methylation analysis of the *PGRMC1*, *UNC119B*, *FNDC3B*, and *RERE* genes in low-dose irradiated HAECs. **A** Schematic representation of the CpG island structures within the promoter regions of *PGRMC1*, *UNC119B*, *FNDC3B*, and *RERE*. Amplicon for MSP analysis is indicated with a green bar. **B** Quantitative MSP of the four genes in irradiated HAECs and controls. All quantitative methylation levels were normalized by the *Alu* element. Statistical analysis data are presented as mean ± SD, black dots indicate replicates (*n* = 4). *p < 0.05; **p < 0.01; ***p < 0.01 compared with control

**Fig. 5:**
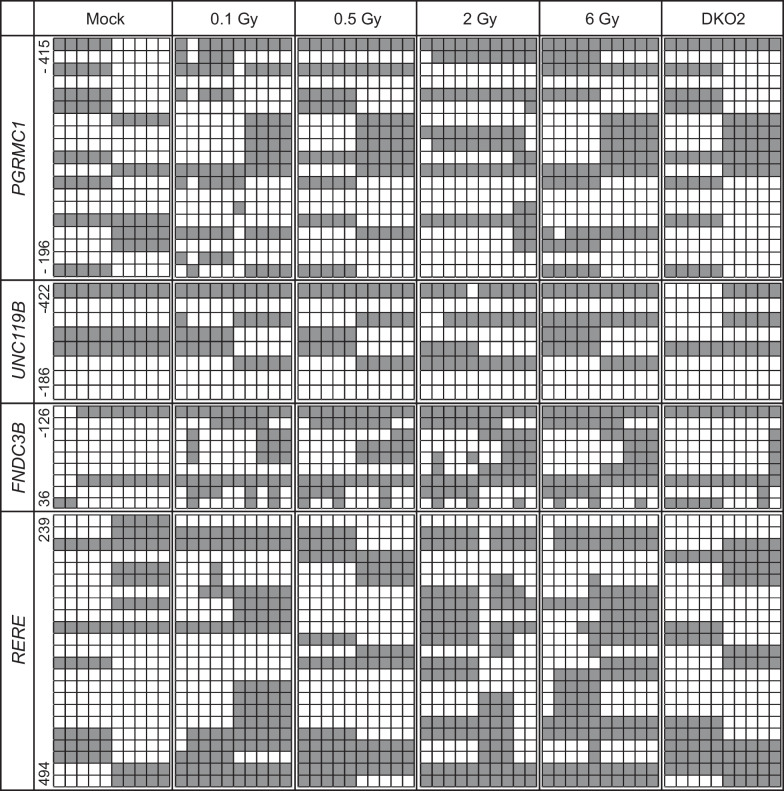
Bisulfite sequencing analysis of CpG islands in the promoter regions of the candidate genes. Bisulfite sequencing analysis was performed with irradiated HAECs. The location of CpG sites in the *PGRMC1* (upstream region from − 415 to − 196) relative to the TSSs of exon 1 are *UNC119B* (upstream region from − 422 to − 186), *FNDC3B* (upstream region from − 126 to + 36), and *RERE* (upstream region from + 239 to + 494), where each box represents a CpG dinucleotide. Black boxes represent methylated cytosines, and white boxes represent unmethylated cytosines

While low-dose radiation can potentially trigger widespread alterations in DNA methylation, the DNA methylation profiles of only a limited number of genes in HAECs and HCAECs are available for further investigation, which makes it challenging to confirm whether these genes are actually affected by promoter DNA methylation after exposure to low-dose irradiation in HAECs or HCAECs. Thus, to experimentally validate whether these genes were regulated by promoter methylation changes, we used several strict criteria. Candidate genes should have (1) a typical CpG island in the promoter region for methylation, (2) the pattern of promoter methylation should be negatively correlated with its transcriptional expression, and (3) increase of DNMT1 distribution in the promoter regions of candidate genes upon low-dose irradiation. Based on these criteria, no genes were available for further methylation analysis of low-dose (0.1 Gy)-irradiated HCAECs. However, nine genes from the DNA methylation profiles of 0.1 Gy-irradiated HAECs were available for experimental validation. We first aimed to validate whether these genes were hypermethylated in 0.1 Gy-irradiated HAECs compared with those in the control cells. For this analysis, we designed MSP primers located within the CpG islands of these genes and conducted an MSP analysis (Additional file 1: Table S1). Subsequently, we conducted an extensive conventional MSP analysis to examine the promoter methylation patterns of the candidate genes in both the control and irradiated HAECs. We examined the CpG islands of four genes (*PGRMC1*, *UNC119B*, *FNDC3B*, and *RERE*) in the UCSC database and found that a typical CpG island was located in the promoter region upstream of these genes (Fig. 4A). According to the DNA methylation profile, methylation was undetectable in the four genes (*β*-value = 0) but was significantly increased (*β*-value = 1) in 0.1 Gy-irradiated HAECs. To validate these data, we performed quantitative MSP analysis in irradiated HAECs and compared them with controls. Notably, four genes exhibited a significant increase in methylation levels in irradiated HAECs when compared with those in the control group. Remarkably, this increase in methylation commenced at low doses of radiation (0.1 Gy) in HAECs, implying that even low-dose radiation was sufficient to trigger promoter hypermethylation of these genes in HAECs (Fig. 4B). Next, we confirmed the methylation levels of these four genes at the DNA sequence level using bisulfite sequencing analysis, which revealed denser methylation of the four genes in irradiated HAECs compared with that in the controls. These findings strongly indicate that the DNA methylation levels of these four candidate genes increased in the irradiated HAECs compared to the control (Fig. 5).

**Fig. 6:**
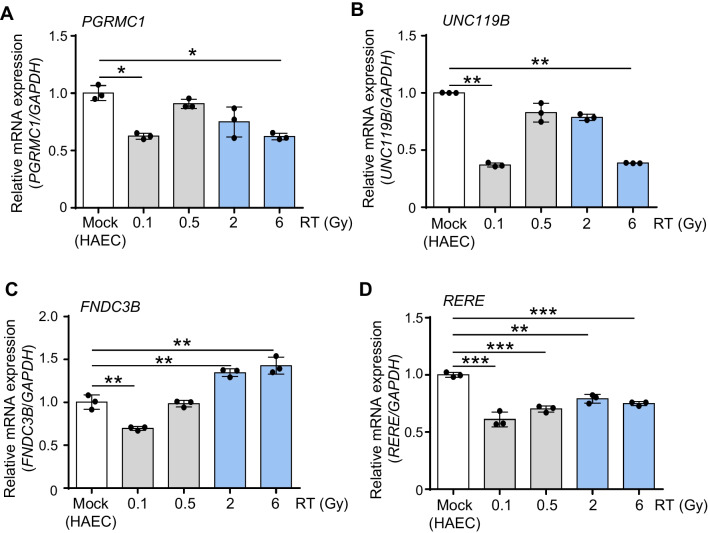
Verification of the changes in the expression of candidate genes in irradiated HAECs using qRT-PCR. **A**–**D** qRT-PCR analyses of *PGRMC1*, *UNC119B*, *FNDC3B*, and *RERE* in irradiated HAECs and controls. Human *GAPDH* was used for normalization of expression levels. Statistical analysis data are presented as mean ± SD, black dots indicate replicates (*n* = 3). *p < 0.05; **p < 0.01; ***p < 0.01 compared with control

To correlate promoter hypermethylation with the expression of these four genes in irradiated HAECs, we examined their transcriptional expression using qRT-PCR. Gene expression levels significantly decreased in HAECs irradiated with low-dose radiation (0.1 Gy) compared to those in the control group (Fig. 6). Our data suggest that low-dose radiation can effectively decrease gene expression by inducing promoter hypermethylation, confirming that these genes are strong candidates for hypermethylation induced by low-dose radiation.

### Low-dose radiation-induced hypermethylated genes correlate with increasing levels of DNMT1 in their promoter regions

**Fig. 7:**
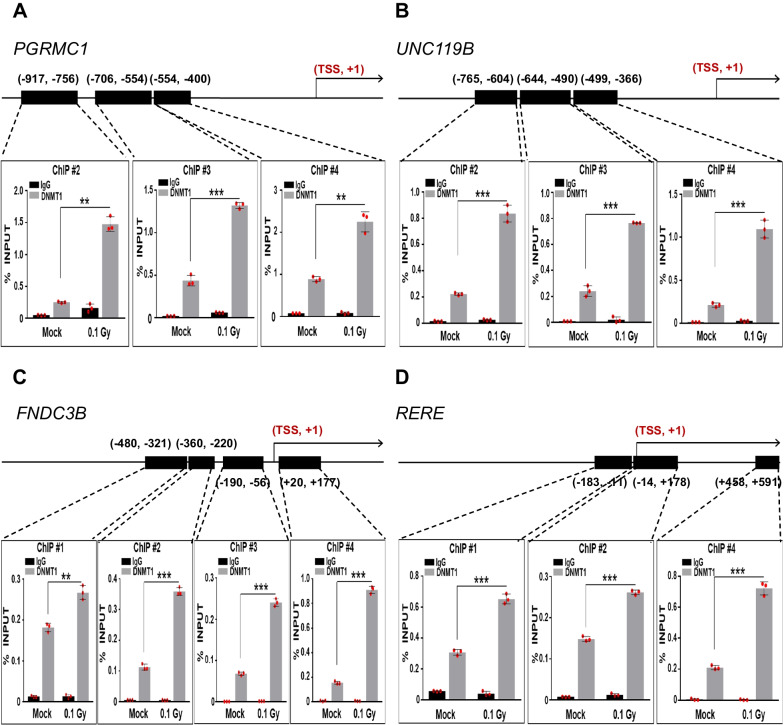
Association between DNMT1 enrichment and the promoter regions of candidate genes in HAECs exposed to low-dose radiation (0.1 Gy). **A**–**D** ChIP assays were performed to determine the levels of DNMT1 at the promoter regions of candidate genes (*PGRMC1*, *UNC119B*, *FNDC3B*, and *RERE*) in HAECs exposed to low-dose radiation (0.1 Gy). *GAPDH* was used as a negative control. Cross-linked and sheared chromatin was immunoprecipitated with an anti-IgG antibody (black bar) or with anti-DNMT1 (light grey bar). The results are presented as a percentage of the input chromatin, and the quantification of the associated chromatin in the ChIP samples was performed using qRT-PCR. Statistical analysis data are presented as mean ± SD, red dots indicate replicates (*n* = 3). *p < 0.05; **p < 0.01; ***p < 0.001; *TSS* transcription start site

The expression of many proteins associated with DNA methylation, including DNMT1, increased in irradiated HAECs and HCAECs (Fig. 1). DNMTs are enzymes that are crucially involved in a key step in the DNA methylation process [17]. DNMT1 is predominantly responsible for DNA methylation in cancer cells [28, 29] and plays a pivotal role in sustaining aberrant promoter methylation. Having identified several hypermethylated genes induced by low-dose radiation in HAECs, we investigated whether these genes might have a direct association with the DNMT1 protein in terms of methylation changes following low-dose irradiation. Using ChIP assay, we examined the correlation between DNMT1 and CpG islands in the promoter region of each candidate gene in 0.1 Gy-irradiated HAECs. We observed that DNMT1 levels were significantly increased in the promoter regions of most candidate genes (Fig. 7). These data provide robust evidence supporting a correlation between increased DNMT1 levels and DNA hypermethylation within the promoter regions of candidate genes, which, in turn, leads to decreased gene expression. Collectively, our validation strategy strengthens the notion regarding the potential biological significance of these newly identified genes that induce hypermethylation in response to low-dose radiation in HAECs. These findings suggest novel epigenetic functions of these previously unexplored genes in normal ECs.

## Discussion

Industrial and scientific advances have increased the potential for radiation exposure across various facets of daily life. Concerns related to occupational radiation exposure, the trajectory of nuclear power, manned space exploration, and radiological terrorism underscore the need for a comprehensive understanding of the health hazards linked to low-dose radiation exposure [6]. Additionally, individuals are exposed to low-dose radiation during medical procedures for diagnostic purposes. According to the guidelines set forth by the ICRP, individuals at risk of recurrent radiation exposure include healthcare and nuclear industry professionals. These individuals are subject to monitoring and dose limitations, with a recommended yearly limit of 20 mSv and a maximum allowable exposure of 50 mSv in any single year, as well as an overall cap of 100 mSv every 5 years [9, 30].

DNA methylation, an epigenetic alteration, plays a pivotal role in an extensive array of biological processes, including embryogenesis [31], X-chromosome inactivation [32], genomic imprinting [33], cell differentiation [34], inhibition of transposable elements [35], and cognitive functions, such as learning and memory [36]. To carry out its functions in various processes, DNA methylation undergoes dynamic and regionally regulated changes in response to both internal and external signals. In this context, numerous studies have been performed to uncover the influence of IR on DNA methylation [37–40]. A common trend observed across multiple studies indicates that radiation frequently triggers localized hypermethylation of CpG islands while simultaneously inducing global hypomethylation of DNA [19, 41]. This similarity to the changes in DNA methylation observed during tumor development has sparked speculation about the potential contributions of radiation-induced CpG island hypermethylation to gene silencing and the role of global DNA methylation loss in promoting tumorigenesis [39]. However, most of the aforementioned studies have focused on changes in DNA methylation following radiation doses exceeding 100 mSv. To the best of our knowledge, Newman et al. [42] investigated alterations in DNA methylation in mice after a single low-dose X-ray exposure of 10 mGy.

Here, DNA methylation profiling was used to explore the potential influence of low-dose radiation on the initiation of endothelial dysfunction. We examined the DNA methylation profile of ECs in response to low-dose radiation under normal conditions. This is because EC dysfunction is a significant risk factor for both cardiovascular diseases and diabetes mellitus [43]. We performed DNA methylation arrays using HAECs and HCAECs exposed to 100 mGy of low-dose radiation compared with higher doses (2 and 6 Gy) and controls. We also listed the genes potentially associated with endothelial damage. Our findings indicate that the exposure of normal ECs to low-dose radiation induces a widespread hypermethylation pattern, suggesting that even low-dose radiation can sufficiently trigger alterations in DNA methylation. Additionally, we confirmed the hypermethylation of several particularly intriguing genes in response to alterations employing multiple experimental validations, highlighting their potential as biomarkers for assessing the risks associated with low-dose radiation exposure.

In this study, a genome-wide DNA methylation array was useful to identify new genes induced by hypermethylation in response to low-dose radiation. We emphasize that these genes were newly discovered to be hypermethylated in response to low-dose IR, a correlation that was corroborated through various experimental approaches, including qRT-PCR and MSP analyses. Additionally, we conducted ChIP assays to investigate the interaction between DNMT1 and the promoter regions of candidate genes following low-dose IR treatment, providing further evidence for the transcriptional regulation of these genes by DNMT1. The presence of DNMT1 preference for methylated substrates, with a 5- to 30-fold bias, led to its recognition as the enzyme responsible for preserving methylation patterns after DNA replication. Indeed, genetic knockout of DNMT1 in human cells results in an abnormal nuclear structure and disrupts the distribution of heterochromatin protein 1 [44]; this underscores the strong connection between these two processes. Subsequently, DNMT3a and DNMT3b were identified through EST database searches and proposed as enzymes responsible for de novo methylation [31].

Progesterone (P4) receptor membrane component 1 (PGRMC1) is a member of the membrane-associated P4 receptor (MAPR) family and belongs to the cytochrome b5 (cytb5) protein group. This versatile protein performs a wide array of functions [45, 46]. Human PGRMC1 contains predicted binding site motifs for *Src homology 2* (SH2) and *Src homology 3* (SH3) domain-containing proteins. In addition, it features multiple phosphorylation sites, including *s57, t178*, and *s18*1, which are believed to modulate its activity [47]. On the folded protein surface, there is a juxtaposition of the SH3 target motif adjacent to s57, along with the SH2 target motifs containing Y139 and T178/Y180/S181. This arrangement forms a potential proximity-stimulated tripartite signaling platform [47, 48]. Consequently, PGRMC1 serves as a key factor and an influential participant in numerous cellular signaling pathways that regulate cell growth and proliferation. To our knowledge, this study represents a pioneering effort in identifying four genes (*PGRMC1*, *UNC119B*, *RERE*, and *FNDC3B*) as candidate methylation biomarkers for assessing the risk of low-dose radiation exposure.

We conducted a thorough literature search to identify the biological functions of confirmed low-dose radiation-induced hypermethylation. UNC119 is a myristoyl-binding protein that facilitates the intracellular transportation of myristoylated cargo proteins to their respective functional destinations [49]. The human *UNC119* was initially discovered to be enriched in the retina and was designated as human retina gene 4 (*HRG4*) [50]. Truncation mutations in *UNC119* are detected in human patients and can lead to retinal degeneration in transgenic mice [51, 52]. Mammalian genomes harbor two *UNC119* genes, *UNC119A* and *UNC119B*, although the functional distinctions between these two genes have yet to be fully elucidated.

*RERE* is prominently expressed in the brain and has been associated with numerous single-nucleotide polymorphisms (SNPs) identified in genome-wide association studies (GWAS) related to various brain disorders [53–55]. The protein encoded by *RERE* plays a crucial role in transcriptional repression during embryonic development, chromatin remodeling, and cell survival. Recently, the interaction of *RERE* with G9A, a histone methyltransferase known for its involvement in transcriptional repression, suggested that *RERE* may play a significant role in gene regulation.

Fibronectin type III domain containing 3B (*FNDC3B*), also known as a factor in adipocyte differentiation 104 (*FAD104*), was initially recognized as a regulator of adipocyte differentiation [56]. Subsequent gene-targeting studies provided evidence that *FNDC3B* plays a role in cell proliferation, adhesion, spreading, and migration in *FNDC3B*-deficient mice [57]. *FNDC3B* has also been identified as an oncogene that promotes cell migration in hepatocellular carcinoma [58, 59]. However, the prognostic significance and functional role of *FNDC3B* in cancer remain unexplored.

Recently, Lee et al. [60] reported the potential impact of *PGRMC1* on cardiac metabolism under energy-deficient conditions. Their data indicate that PGRMC1 apparently regulates cardiac metabolism by influencing the balance between glucose and fatty acid utilization based on nutritional status and nutrient availability in the heart. However, the roles of *UNC119B, RERE,* and *FNDC3B* in cardiovascular diseases are not known. Our study is the first to report the association of epigenetic changes in these genes with cardiovascular diseases. According to network analysis using the STRING software, we identified a possible biological network of the four gene candidates related to low-dose irradiation. Each gene was associated with various genes involved in diverse biological functions (Additional file 1: Fig. S2). Recently, another research group explored changes in DNA methylation in the blood of healthy individuals exposed to CT radiation. Their findings suggested that there were no significant alterations in genome-wide DNA methylation levels [61]. In line with the impact of low-dose radiation on ECs, Lee et al. recently reported the impact of low-dose radiation (less than 100 mGy) on ECs in both diabetic and non-diabetic conditions, focusing on their role in cardiovascular health using RNA sequencing. Their findings suggested a potential link between low-dose radiation and cardiovascular diseases [62]. Expanding upon insights from prior studies, our data strongly reinforce the notion that low-dose radiation can trigger molecular changes in ECs, particularly within the domain of epigenetic modifications, including DNA methylation. Through a comprehensive validation process, we identified several genes that have the potential to serve as biomarkers for detecting the risks associated with low-dose exposure.

## Conclusion

In summary, we explored the effects of low-dose radiation on DNA methylation profiles in immortalized normal HAECs and HCAECs. We demonstrated that low-dose radiation can sufficiently induce global DNA methylation changes in terms of the levels of key proteins associated with DNA methylation. Consistent with these data, using a DNA methylation array, we further verified that low-dose radiation induces global DNA methylation changes. Furthermore, we identified genes whose promoters exhibited hypermethylation induced by low-dose radiation, which led to reduced transcriptional gene expression. We also validated these promoter methylation changes at the genomic level using bisulfite sequencing. We observed an increase in the level of DNMT1 in the promoter regions of these genes in low-dose irradiated HAECs compared to controls. Taken together, our data strongly suggest that low-dose radiation can effectively induce alterations in DNA methylation in normal ECs. This discovery led us to propose that these newly identified genes could serve as valuable DNA methylation biomarkers for detecting the risk of radiation exposure or the development of diseases, including cardiovascular diseases.

### Supplementary Information

**Additional file 1**. **Table S1.** Primer list for this study. **Fig. S1.** IR induces changes in the levels of epigenetic regulators in HAECs and HCAECs. (A-B) Western blots show the protein expression levels of methylation regulatory factors (DNMT1, MBD2, MBD4, and MeCP2) in primary HAECs and HCAECs irradiated with various doses of radiation ranging from 2 to 8 Gy and a control. The blots were probed using anti-DNMT1, anti-DNMT3b, anti-DNMT3A, anti-DNMT3L, anti-MBD4, antiMBD-2, and anti-MECP2 antibodies. Beta-actin was used for normalization in western blotting analyses. **Fig. S2.** Network analysis of target genes using STRING software. Four separate network analyses were conducted with each of the following genes (in black): *PGRMC1, UNC119B, FNDC3B*, and *RERE*. Genes exhibiting hypermethylated CpGs (showing at least a 0.1 β-value increase compared to controls, which received no IR treatment) and those located near the TSS are marked in orange. Similarly, genes exhibiting hypomethylated CpGs (showing at least a 0.1 β-value decrease compared to controls) and those located near the TSS are marked in blue. Genes with no significant difference in β-value are indicated by grey circles. TSS, transcription start site.

## Data Availability

The datasets generated and/or analyzed during the current study are available in the gene expression omnibus (GEO) repository, [https://www.ncbi.nlm.nih.gov/geo/query/acc.cgi?acc=GSE246076].

## Abbreviations

EC: Endothelial cell
IR: Ionizing radiation
HAEC: Human aortic endothelial cell
HCAEC: Human coronary artery endothelial cell
MSP: Methylation-specific polymerase chain reaction
qRT-PCR: Quantitative real-time reverse transcription polymerase chain reaction
SD: Standard deviation
TSS: Transcription start site
ChIP: Chromatin immunoprecipitation
