# Supplementary material for: Identification of DNA methylation biomarkers for evaluating cardiovascular disease risk from epigenome profiles altered by low-dose ionizing radiation
Source: Clin Epigenetics. 2024 Feb 1;16:19. doi: 10.1186/s13148-024-01630-0 (PMC10835887; doi:10.1186/s13148-024-01630-0)
Supplement: Supplementary file 1 — Additional file 1. Table S1. Primer list for this study. Fig. S1. IR induces changes in the levels of epigenetic regulators in HAECs and HCAECs. (A-B) Western blots show the protein expression levels of methylation regulatory factors (DNMT1, MBD2, MBD4, and MeCP2) in primary HAECs and HCAECs irradiated with various doses of radiation ranging from 2 to 8 Gy and a control. The blots were probed using anti-DNMT1, anti-DNMT3b, anti-DNMT3A, anti-DNMT3L, anti-MBD4, antiMBD-2, and anti-MECP2 antibodies. Beta-actin was used for normalization in western blotting analyses. Fig. S2. Network analysis of target genes using STRING software. Four separate network analyses were conducted with each of the following genes (in black): PGRMC1, UNC119B, FNDC3B, and RERE. Genes exhibiting hypermethylated CpGs (showing at least a 0.1 β-value increase compared to controls, which received no IR treatment) and those located near the TSS are marked in orange. Similarly, genes exhibiting hypomethylated CpGs (showing at least a 0.1 β-value decrease compared to controls) and those located near the TSS are marked in blue. Genes with no significant difference in β-value are indicated by grey circles. TSS, transcription start site. [file 13148_2024_1630_MOESM1_ESM.pdf]

Table S1. Primer list for this study

Genes	Targets	Forward primer (5' - 3')	Reverse primer (5' - 3')
PGRMC1	Unmeth	GATTTTAGTAATTTTGGGGGTGTT	AAAAACAAAAAACTCATTCTTCAAA
	Meth	ATTTTAGTAATTTCGGGGGTGTC	AAAAACAAAAAACTCATTCTTCGAA
	RT-PCR	GTGCACCAAGAGCAAAGGAT	GGACAACAGTGCAGTCCAGA
	BS-Seq	GGGAAAAGTTTGTAGTAATAGAAAGG	CAATTCTACCTAAAAAACCCCAAC
	ChIP-2	AGGCCTAGGATCCTTCCAAA	TCACCAGGCATTAACAAGCA
	ChIP-3	TTCGGAGAACAAACCCAAAG	TCATTTTCACCTTGGGAACC
	ChIP-4	GGTTCCCAAGGTGAAAATGA	CACCGACTCCCTTTCTGTTG
UNC119B	Unmeth	TAGTAGTATTTTTTGAATGGATTGA	AAAAACTATAAAATTCCCTTTCACA
	Methy	TAGTAGTATTTTTTGAATGGATCGA	AAAAACTATAAAATTCCCTTTCGC
	RT-PCR	GCTTCTGTTCCTTTGCCTTG	CAGGAAGCACATGCTTGAAA
	BS-Seq	TGGTAGGATTAAAGTTGGGGTAGTA	ACCATCAACAACCTAAAATACACATAA
	ChIP-2	AGGGTTGTTGTGAGGTGAGG	GAATCTTCCGTGCACCATCT
	ChIP-3	TGTTGGGACTGGTGACAGAA	ATCAGACCCCCAAGATCCTC
	ChIP-4	AACATCGTGGAGGATCTTGG	CCTAACTGGGTGACTGCACA
FNDC3B	Unmeth	TTTTTGTTTAGTTAGGTAGGGTTGG	AAAAAATTTAATAAAAAATTCCACC
	Methy	GAGTTTTTGTTTAGTTAGGTAGGGTC	AAAAAAATTTAATAAAAAATTCCGC
	RT-PCR	AGTCTCCCTGTTCGCACACT	CTCTGGGCCATGGTACACTT
	BS-Seq	GAGTTTTTGTTTAGTTAGGTAGGGT	CCAAAAAAAATTTAATAAAAAATTCC
	ChIP-1	CTGCAGTCCCCAAAACAAAC	TTGTTAACCCCCACAGCAAC
	ChIP-2	TGGGGGTTAACAACAACAAAA	TCCTTTTGGTTCAGCCAGAC
	ChIP-3	GTCTGGCTGAACCAAAAGGA	CTACCAGGAGGGTTTGATGG
	ChIP-4	CCATCAAACCCTCCTGGTAG	ACCTTCCCTAGGGCTGAAAC
RERE	Unmeth	TATTTTATTATTGATGGTGTAGTGT	CCAAACAAATAACTACTTTCACAAC
	Methy	TATTTTATTATTGATGGTGTAGCGT	CGAACGAATAACTACTTTCACGAC
	RT-PCR	AGTGAAGAAGTCGGCCAAGA	TCTTTGGGGTCACTGCTACC
	BS-Seq	GGTTTTTGTTTTTGAAAGATTTGTT	ATTAAACACTTACCTTCCCCTAAAAC
	ChIP-1	AAAAGAGATCCCGCCTCCT	AGCGTCTGTGGGAACAGC
	ChIP-2	GCCGCTGTTCCCACAGAC	AATCACCATCATCCTCAGGG
	ChIP-4	GATGGTGTAGCGCTTTAGGG	ACCCCAGCCTTTTCTTCCT

Figure S1.

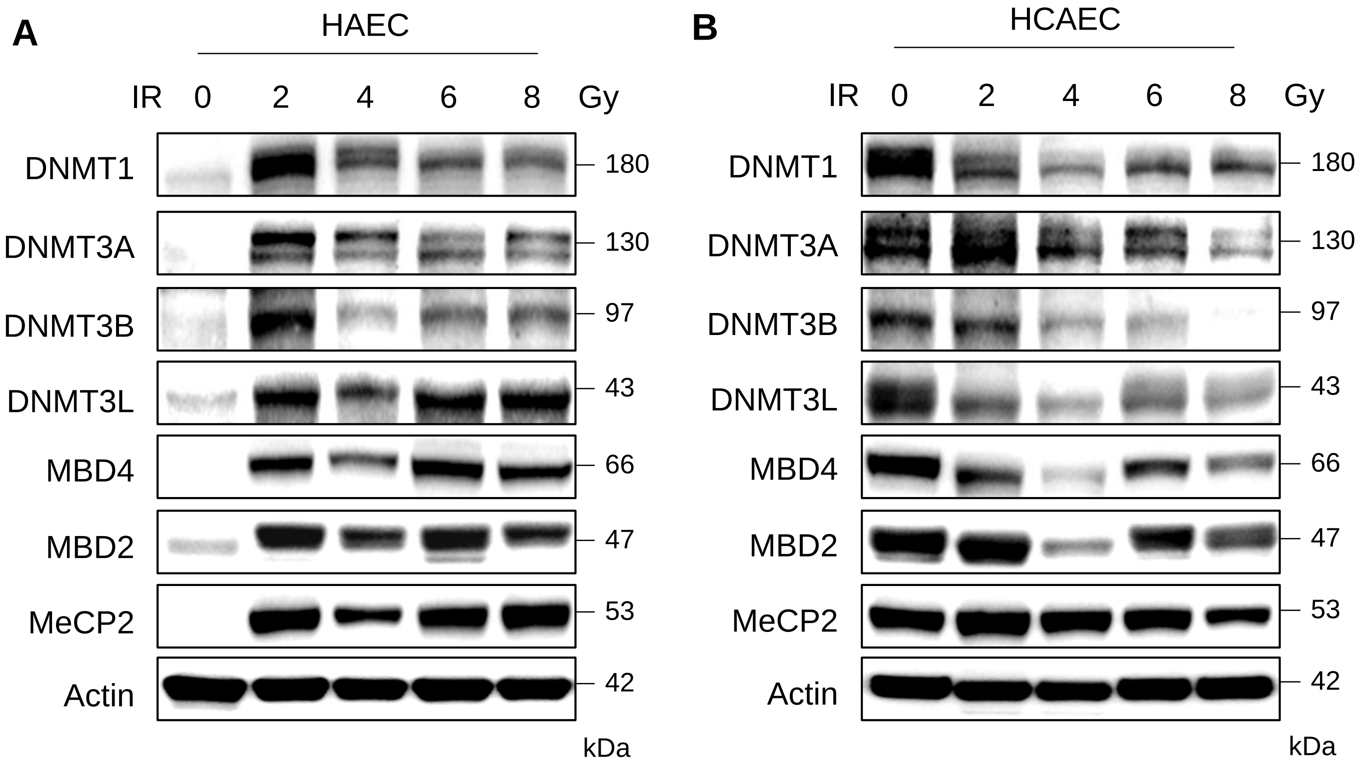
A
HAEC
IR 0 2 4 6 8 Gy
DNMT1
DNMT3A
DNMT3B
DNMT3L
MBD4
MBD2
MeCP2
Actin
180
130
97
43
66
47
53
42
kDa
B
HCAEC
IR 0 2 4 6 8 Gy
DNMT1
DNMT3A
DNMT3B
DNMT3L
MBD4
MBD2
MeCP2
Actin
180
130
97
43
66
47
53
42
kDa

Figure S2.

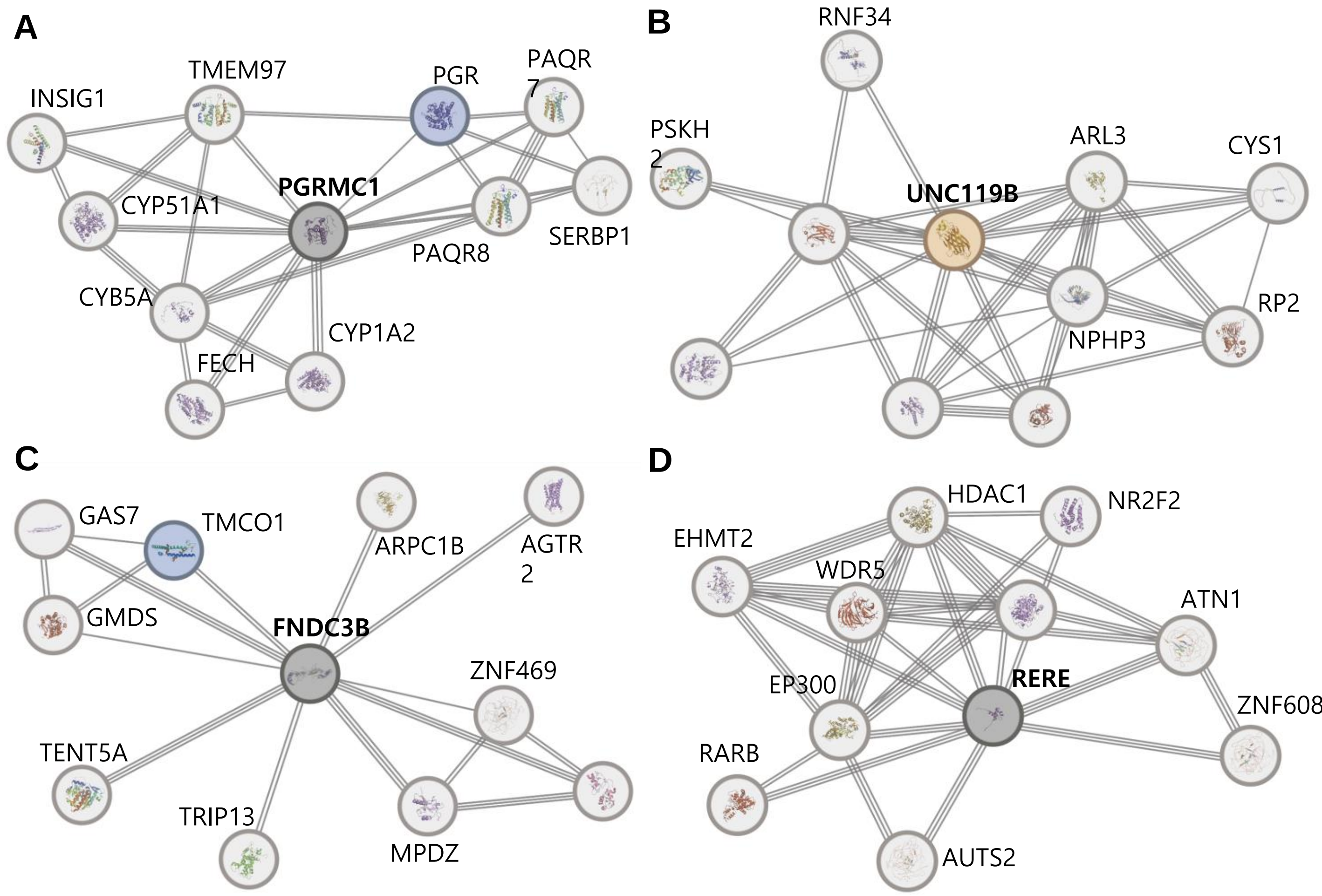
A
INSIG1
TMEM97
PGR
PAQR
7
CYP51A1
PGRMC1
SERBP1
PAQR8
CYB5A
CYP1A2
FECH
B
RNF34
PSKH
2
ARL3
CYS1
UNC119B
RP2
NPHP3
C
GAS7
TMCO1
ARPC1B
AGTR
2
GMDS
FNDC3B
ZNF469
TENT5A
TRIP13
MPDZ
D
HDAC1
NR2F2
EHMT2
WDR5
ATN1
EP300
RERE
ZNF608
RARB
AUTS2
